# The role of inorganic nitrogen in successful formation of granular biofilms for wastewater treatment that support cyanobacteria and bacteria

**DOI:** 10.1186/s13568-017-0444-8

**Published:** 2017-07-10

**Authors:** Kristie Stauch-White, Varun N. Srinivasan, W. Camilla Kuo-Dahab, Chul Park, Caitlyn S. Butler

**Affiliations:** Department of Civil and Environmental Engineering, University of Massachusetts, Amherst, 01003 USA

**Keywords:** Granular biomass, Filamentous cyanobacteria, Wastewater treatment, Inorganic nitrogen, Oxygenic photogranules

## Abstract

Recently, the use of phototrophs for wastewater treatment has been revisited because of new approaches to separate them from effluent streams. One manifestation uses oxygenic photogranules (OPGs) which are dense, easily-settleable granular biofilms of cyanobacteria, which surrounding populations of heterotrophs, autotrophs, and microalgae. OPGs can remove COD and nitrogenous compounds without external aeration. To better grow and maintain biomass in the proposed wastewater process, this study seeks to understand the factors that contribute to successful granulation. Availability of initial inorganic nitrogen, particularly ammonium, was associated with successful cultivation of OPGs. In the first days of granulation, a decrease in ammonium coupled with an increase in a cyanobacterial-specific 16S rRNA gene, may suggest that ammonium was assimilated in cyanobacteria offering a competitive environment for growth. Though both successful and unsuccessful OPG formation demonstrated a shift from non-phototrophic bacterial dominated communities on day 0 to cyanobacterial dominated communities on day 42, the successful community had a greater relative abundance (46%) of OTUs associated with genera *Oscillatoria* and *Geitlernema* than the unsuccessful community (27%), supporting that filamentous cyanobacteria are essential for successful OPG formation. A greater concentration of chlorophyll *b* in the unsuccessful OPG formation suggested a greater abundance of algal species. This study offers indicators of granulation success, notably availability of inorganic nitrogen and chlorophyll *a* and *b* concentrations for monitoring the health and growth of biomass for a potential OPG process.

**Electronic supplementary material:**

The online version of this article (doi:10.1186/s13568-017-0444-8) contains supplementary material, which is available to authorized users.

## Introduction

Cyanobacteria are an extremely diverse phylum and exceedingly adaptive. Within biofilms, there is evidence that they participate in the exchange of oxygen, carbon, and nitrogen species (Abed 2010; Muro-Pastor et al. 2005; Stal 1995). In engineered applications, the oxygenic nature of some cyanobacteria and their symbiosis with other bacterial species make them attractive for biological wastewater treatment. However, separation of filamentous cyanobacteria has been a major barrier to implementation. A potential solution to this issue is the recently cultivated, oxygenic photogranules (OPGs), produced from photo-illuminated activated sludge (Abouhend et al. 2016; Milferstedt et al. in review). OPGs are dense, spherical, interwoven microbial biofilms comprised of cyanobacteria encapsulating heterotrophs, autotrophs, and microalgae. Many successful OPG cultivations have been performed under a variety of conditions and from a variety of municipal waste streams from around the world (Milferstedt et al. in review). In successful cultivations, the presence of motile, filamentous cyanobacteria such as the genera *Oscillatoria*, *Microcoleus*, *Pseudoanabaena*, and *Leptolyngbya,* have been implicated in the structural integrity of the OPG. OPGs remove chemical oxygen demand (COD) and nitrogenous compounds without external aeration by supporting the symbiotic growth of bacteria and phototrophs (Abouhend et al. 2016; Milferstedt et al. in review). Without external aeration, wastewater treatment facilities could save 25–60% of the operating costs of municipal wastewater treatment. Though this is a promising process, the formation and propagation of OPGs within the treatment process is not yet fully understood, which remains a challenge in starting and sustaining the treatment process.

In nature, aggregates of cyanobacteria are known to self-organize into stable population patterns that can support the structure of a biofilm aggregate (Tamulonis and Kaandorp 2014). Although the mechanisms for stimulating the strong granule-like formation is still unknown, studies have pointed to the stickiness of extracellular polymeric substances (Jakub et al. 2013; Tuomainen et al. 2006), filament entanglement (Weber et al. 2007), gliding actions that form cohesive patterns (Tamulonis and Kaandorp 2014), and gliding actions that serve as a defense against extreme light conditions, or predation by microbial grazers (Edyta and Agnieszka 2002). The benefit of sharing of metabolites such as nitrogenous compounds could also initiate granule formation. Spherical aggregates containing phototrophs form in nutrient-limited environments around a concentrations of nutrient-rich materials. One type of cyanobacteria aggregate, cryoconite granules, are found in glacial potholes (Takeuchi et al. 2001). These aggregates are thought to form around nutrients found in dust and debris that collect in cryoconite holes (Segawa et al. 2014). Cyanobacterial, algal and other bacterial species surround fecal pellets within marine environments (Klawonn et al. 2015; Tuomainen et al. 2006). Though some cyanobacteria are capable of nitrogen-fixation, it is energetically intensive. Aggregating and encapsulating a soluble sources of nitrogen species within a diverse community of bacteria and microalgae may help sustain these communities.

Granulation of OPGs has generally been described and the essential role of filamentous cyanobacteria in the parallel structural development under both static and hydrodynamic conditions has been defined (Milferstedt et al. in review). However, on occasion, granulation does not proceed successfully. The environmental conditions and ecological factors that differentiate a successful and unsuccessful granulation are still unclear. The contribution of the initial nutrients, particularly nitrogen content and speciation within the local environment, may be an integral factor. In an effort to explore the association of initial nitrogen in successful cultivation, we present a study of static cultivation for both successful and unsuccessful OPG formation. The progression of granulation is considered through the evaluation of soluble substrates, relative abundance of key functional genes, and microbial ecology for successful and unsuccessful scenarios. Understanding the conditions that lead to successful granulation is critical for consistently growing OPG biomass for both seeding reactors and sustaining new biomass growth within operating reactors.

## Materials and methods

### Cultivation

Activated sludge from aeration basin at the Amherst Wastewater Treatment Plant in Amherst, Massachusetts was collected, and 10 mL well-mixed aliquots were pipetted into 20 mL scintillation vials and capped immediately after returning to the lab, leaving head space. Additional activated sludge was used for day 0 chemical analysis and DNA-based molecular methods and t = 0 data represents initial conditions of the experiment and water quality for the activated sludge. Vials were kept in static conditions, illuminated under broadband fluorescent lights at approximately 10 klux, 24 h per day at room temperature. Sampling was done every 2–3 days during the first 2 weeks, and then, weekly for 42 days. At day 42, the cultivation was considered complete and a final sample collection was performed. To determine the success of granulation (>50% of vials at the end of cultivation yielded a granule, n = 10–20), a shake test was performed on the remaining vials by using three firm vertical shakes by hand and then observing the vial contents. When a granule remained intact with little to no cloud of particulates in the bulk liquid, granulation was determined to be successful. Five cultivations were performed over the period of 1 year. Each cultivation consisted of 200–300 vials to allow for the destructive sampling of 2–3 vials for each analysis at each sampling point. Photographic and microscopic record was collected throughout the cultivation. Photographs were collected at each sampling time for triplicate sample vials. Microscopy was performed on an EVOS FL Color AMEFC 4300 light microscope by sectioning granules and mature biomass on day 42 in a petri dish with a razor blade and placing sampled sections on a microscope slide at beginning and end of each cultivation. At least three selections from each section were inspected. Photos of communities were taking at 2× and 40× magnification in multiple locations for each sample.

### Chemical analysis

For soluble chemical analysis, each sample was filtered through 0.2 µm syringe filters and stored at −20 °C for subsequent analysis. Nitrate, nitrite, phosphate, chloride, and sulfate were measured using a Metrohm 850 Professional Ion Chromatograph (IC) (Metrohm Inc., Switzerland) with a Metrosep A Supp 5-250 anion column (Metrohm Inc., Switzerland. For anion analysis, 3.2 mM Na_2_CO_3_, 1.0 mM NaHCO_3_ eluent was pumped at 2.6 mL/min, with a 100 mM HNO_3_ suppressor solution and using a 20 µL sample loop. Ammonium was monitored using a Metrosep C 2-250 cation column (Metrohm Inc., Switzerland). For cation analysis, an eluent consisting of 0.75 mM dipicolinic acid and 4 mM tartaric acid was pumped at 1 mL/min, using a 10 µL sample loop. Soluble nitrogen and COD were measured using Hach kits (Hach, Loveland, CO, USA), per standard methods 10,023, 10,071, 8000. Total nitrogen was measured with Hach kits following manufacturer’s direction and the organic nitrogen was calculated as the total nitrogen less the inorganic species. Chlorophyll, total suspended solids (TSS) and volatile suspended solids (VSS) were measured using standard methods 10020, 208E (APHA 2005). pH was measured using a Fisher-Scientific probe.

### DNA extraction

Total microbial DNA was extracted using a MoBio PowerSoil DNA Extraction kit following manufacturer instructions and using 0.08 g of granule biomass. DNA was extracted from three vials on each sampling day following 10 s of homogenization (IKA T18 basic ULTRA-TURRAX homogenizer) and dewatering by centrifuge and pipetting. DNA quantity and quality was determined by Nanodrop. Concentrations of DNA extract that met minimum quality standards (~1.8 for 260 nm/280 nm and ~2 for 260 nm/230 nm) varied between 5 and 50 ng/µL.

### PCR and quantitative PCR

Functional marker gene analyses were performed on either an Applied Biosystems Step One or an MJ Research quantitative PCR system. qPCR reactions were done in 25 µL reactions using 12.5 µL iTaq 2× Universal SYBR Green Supermix, 0.2 µM each forward and reverse primers and 10 ng template DNA. Real-time amplification was performed at 95 °C for 10 min. followed by 40 cycles consisting of 95 °C for 15 s, T_annealing_ for 1 min., and 72 °C for 30 s, followed by 30 s at 55 °C and a +0.3 °C ramp up every 15 s to 95 °C to determine the melting curve for the target genes with the appropriate the annealing temperature listed for each primer. Target genes were *amoA* − T_annealing_ = 58 °C, (Rotthauwe et al. 1997), *narG* − T_annealing_ = 60 °C, (Bru et al. 2007), Cyanobacterial 16S rRNA gene (given shorthand *CYAN* in this study) − T_annealing_ = 52 °C, (Martins and Vasconcelos 2011), 16S Universal rDNA − T_annealing_ = 60 °C, (Denman and McSweeney 2006) (Additional file 1: Table S1). All reactions were run in triplicate and for two different vials per sampling date—6 qPCR reactions per sampling date. Standard curves, melting curves and negative controls were run for each qPCR run. The relative quantification of primer amplification was analyzed using the comparative C_T_ method (Schmittgen and Livak 2008) using 16S rRNA universal gene as a reference. qPCR reaction efficiency and control sample are reported in the caption of each qPCR figure (Fig. 6; Additional file 1: Figure S6). Statistical analysis of the fold change values for each condition (successful or unsuccessful) was performed using 1-way ANOVA using sampling day as the main effect. Significant interaction effects were further determined with Tukey’s HSD test if the ANOVA results were significant (p < 0.05).

### DNA sequencing and analysis

Extracted DNA from pooled samples extracted in triplicate was sent to Research and Testing Facility (Lubbock, TX) for PCR amplification and sequencing targeting the V4 region using the primers 515F (5′-GTGCCAGCMGCCGCGGTAA-3′) and 806R (5′-GGACTACHVGGGTWTCTAAT-3′) and the amplicons were sequenced on the Illumina MiSeq platform using V3 chemistry. The raw Fastq files were cleaned using Sickle 1.33 (Joshi and Fass 2011) with a minimum window quality score of 20. The quality-controlled sequences were analyzed using mothur (Schloss et al. 2009) using the protocol described in Kozich et al. (2013). The sequences were trimmed to remove primers and barcodes, quality filtered using sickle v1.33 with a minimum quality score of 20, assembled in mothur and aligned to SILVA 123 database. The alignment was screened to remove poorly aligned sequences using vertical = T and trump =  options in mothur. Chimeras were removed using the UCHIME algorithm available through mothur and clustered into OTUs at sequence similarity cutoff of 97% using the average neighbor clustering algorithm. The sequences were classified using the Naïve Bayesian Classifier (80% confidence threshold) using the RDP training set and consensus taxonomy of OTUs was determined using the 80% cutoff. All sequencing data was rarefied (39,038 sequences which is equal to the minimum number of sequences across all samples) to ensure equal number of sequences in each sample prior to all analyses. Other R packages used in the analysis and plotting were ggplot2 (Wickham 2009), dplyr (Wickham and Francois 2015) and ampvis (Albertsen et al. 2015). The top 10 OTUs were compared to all sequences deposited in GenBank using the basic local alignment search tool (BLAST). The phylogenetic tree was constructed using the neighbor-joining method. The evolutionary distances were computed using the Tamura-Nei method. The alignment and evolutionary analyses were conducted in MEGA7 and FigTree v1.4.3 (FigTree 2017) was used for visualization of the tree. The raw paired-end sequencing reads from 16S rRNA gene amplicon sequencing targeting the V4 region are publicly available in the National Center for Biotechnology Information (NCBI) sequence read archive (SRA) under the BioProject accession number PRJNA378555.

## Results

### Soluble nutrient concentration in successful and unsuccessful cultivations

**Fig. 1:**
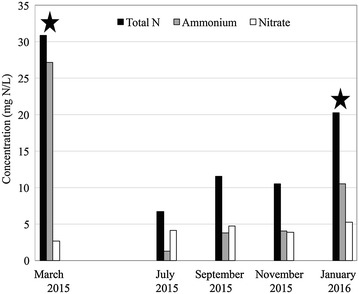
Total soluble initial nitrogen, ammonia, and nitrate concentrations data from the effluent of aeration basin at Amherst wastewater treatment on the day the inoculum biomass was collected for a cultivation. *Stars* indicate the data associated with successful cultivations

**Fig. 2:**
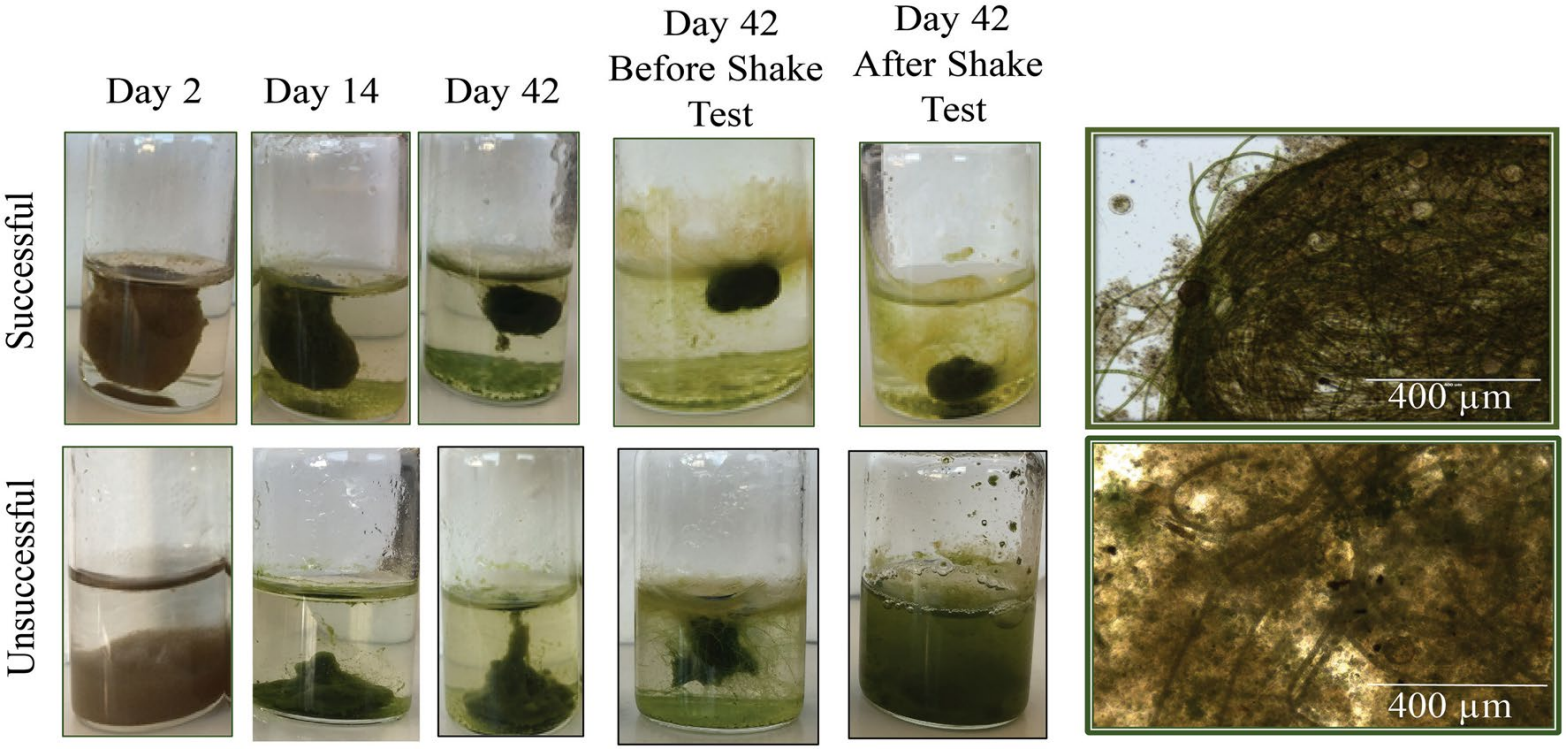
Examples of biomass from successful (*top*) and unsuccessful (*bottom*) cultivation. The results include progression of granulation, before and after the shake test to verify granule rigidity and *bright light* microscopy (×40 objective) of mature OPGs biomass

Over a period of 1 year, five cultivations were performed (Fig. 1). Of the five cultivations, two successfully produced OPGs in over 50% of the vials within 42 days. OPGs produced were between 1 and 2 cm in diameter and were a dense aggregate consistent with OPGs yielded in Milfrestedt et al. in review (Fig. 2). Unsuccessful cultivations also yielded compact, green biomass that in some cases even appeared as spherical OPG aggregates. However, the ‘shake test’ demonstrated that they were loosely aggregated and microscopic images supported a lesser abundance of cyanobacteria on the exterior of the biomass (Fig. 2).

Initial total soluble nitrogen and ammonium concentrations in the aeration basin of the Amherst wastewater treatment plant (WWTP) were greater on collection dates that led to successful granulation (Fig. 1). Initial TSS, total and soluble COD, phosphorus and sulfur data from the treatment process were also considered in potential to produce OPGs but none appeared associated with the success of a cultivation in this study. For consistency in seasonal temperature and wastewater characteristics (Amherst population approximately doubles when the university is in session affecting loading at the WWTP), two case studies representative of the granulation trends for a successful (January 2016) and an unsuccessful cultivation (November 2015) are reported to explore the correlation with initial nitrogen concentration further.

**Fig. 3:**
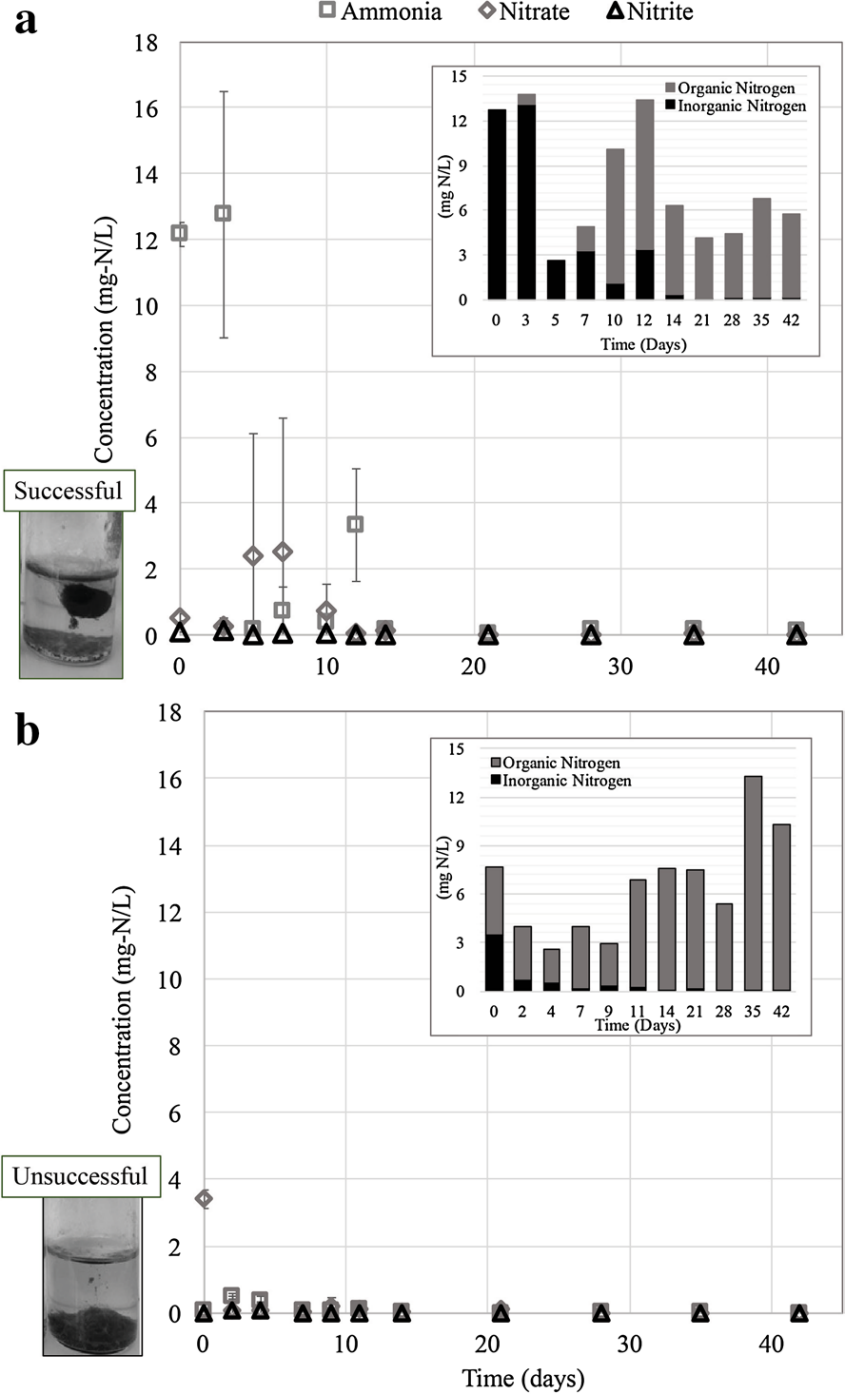
Inorganic nitrogen species for **a** the successful cultivation and **b** the unsuccessful cultivation. The *inset figure* includes the total nitrogen for each sampling point and relative composition of organic and inorganic nitrogen

The availability of inorganic nitrogen, particularly ammonium, appears to be correlated with successful granulation (Fig. 3). Ammonium remained high for the first 2 days of cultivation in the successful granulation and was quickly depleted by day 4 (Fig. 3). The depletion was followed by a corresponding increase of nitrate in solution (2.5 ± 4.1 mg-N/L, ~20% of the initial N). There was high vial to vial variation indicative of differences in granulation progression, however, the overall trends are consistent. The nitrate was depleted by day 12 and remained low for the rest of the cultivation. The depletion of nitrate was followed by increase in ammonium concentration (3.3 ± 1.7 mg-N/L) which decreased within a day. An increase in soluble total organic nitrogen was also observed at this time. This increase could be the result of biomass decay and release of cellular materials. Soluble concentrations of ammonium were low for the rest of the cultivation period. By contrast in unsuccessful cultivation of OPGs, the initial concentration of ammonia was low (<1 mg-N/L) and no corresponding increases in nitrate concentration were observed. Additionally, the successful cultivation ended with a lower total nitrogen concentration than it started, whereas the unsuccessful cultivation resulted in an increase in total soluble nitrogen. The depletion of phosphate from solution was similar for both the successful and unsuccessful cultivations (Additional file 1: Figure S1), though the concentration of phosphate was initially greater for successful cultivations. Soluble COD trends were also similar for both successful and unsuccessful cultivations and increased over the course of the cultivation (Additional file 1: Figure S2). Sulfate concentrations remained constant in both cases throughout cultivation: 34 ± 6.2 mg-S/L for the successful cultivation and 19 ± 3.4 mg-S/L for the unsuccessful cultivation.

### Biomass growth and microbial ecology in cultivation success

**Fig. 4:**
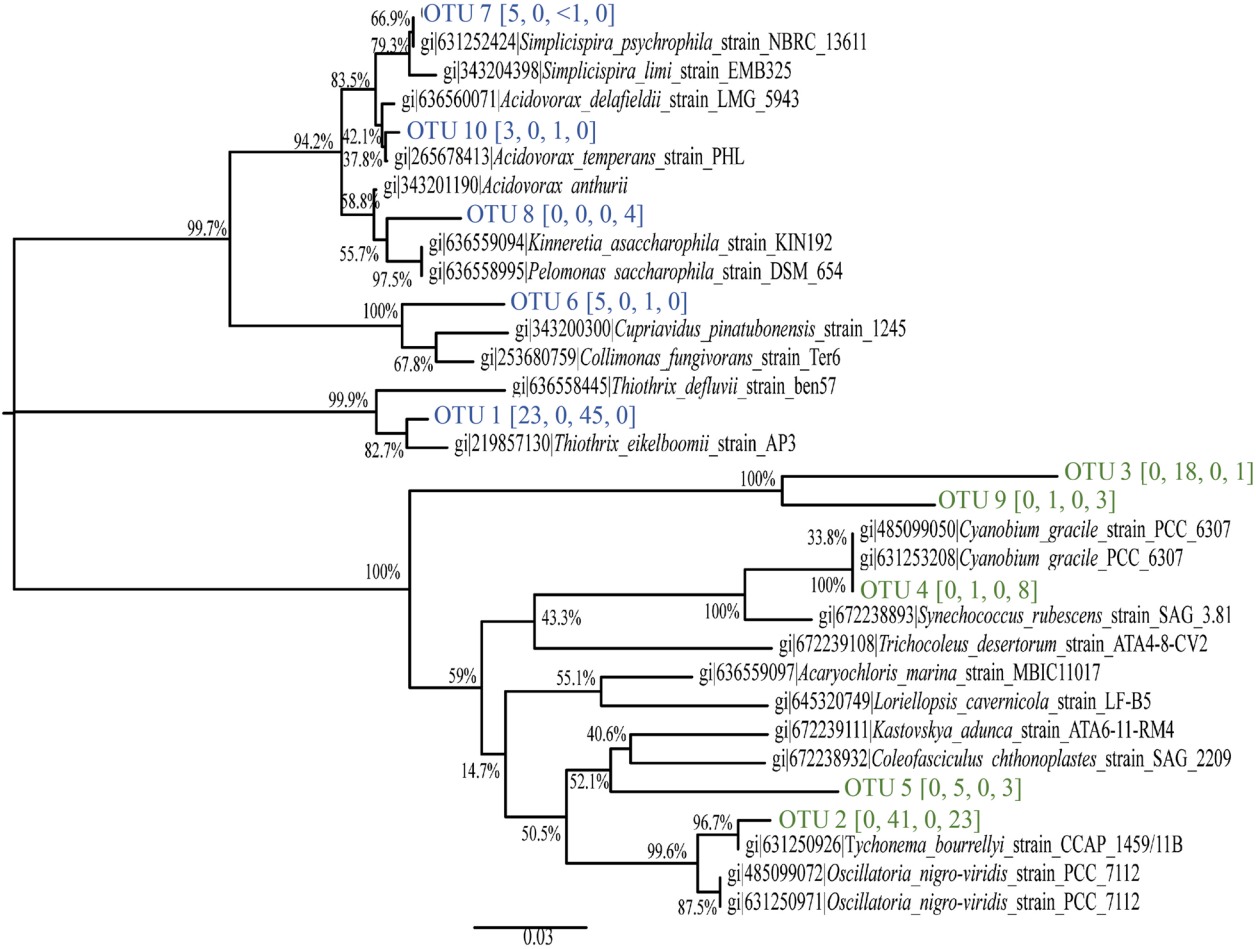
Phylogenic tree of the ten most abundant OTUs found in the initial (t = 0 days) and final (t = 42 days) communities for the successful and unsuccessful cultivation of OPGs. *Blue* associated with bacteria. *Green* associated with cyanobacteria [% succ., 0 day; % succ, 42 days;  %unsucc 0 day, % unsucc, 42 days]

TSS (1740 ± 190 mg-TSS/L) and VSS (1490 ± 150 mg-VSS/L) for successful cultivation were relatively constant throughout the 42 days of incubation but also for unsuccessful cultivation (2040 ± 160 mg-TSS/L, 1750 ± 140 mg-VSS/L). Though the amount of biomass was relatively stable, the community composition demonstrated a dramatic change for both successful and unsuccessful cultivations (Fig. 4). The ecology of initial (day 0) and final community (day 42) were examined for both the successful and unsuccessful cultivations. The 10 most abundant operational taxonomic units (OTUs) based on the 16S rRNA gene were similar for successful and unsuccessful communities but the relative abundances were different. Within these 10 OTUs, the 5 OTUs present in the initial community were undetectable in the final communities and the 5 OTUs that were abundantly present in the final communities were not detected in the initial communities. In the initial communities, OTU 1, putatively assigned to the genus *Thiothrix,* was dominant, though less abundant in the community that successfully produced granules (23%) than the community that did not lead to successful granulation (45%). OTUs 6, 7, and 10 from the phylum Proteobacteria were also slightly more abundant in successful cultivation than the unsuccessful. In particular, OTU 7 (5% in successful and <1% in unsuccessful) most closely matched the genus *Simplicipsira,* which includes microorganisms commonly found in wastewater and are capable of phosphorus removal (*Simplicispira limi)* and denitrification (*Simplicispira psychrophilia)* (Grabovich et al. 2006; Lu et al. 2007) The success of cultivation did not preclude phototrophic growth. The 5 OTUs dominant in the final communities all clustered with cyanobacterial populations. OTU 2 was the most abundant and is related with *Oscillatoria* spp, a filamentous cyanobacteria associated with the structure of mature of oxygenic photogranules (Milferstedt et al. in review). OTU 2 was more abundant in successful cultivation (41%) compared to the unsuccessful cultivation (23%). This was also the case for OTU 5 (5% successful, 3% unsuccessful), putatively assigned to the genus *Geitlernema*, also a filamentous cyanobacteria (Anagnostidis 1989), and OTU 3 (18% successful, <1% unsuccessful). Though OTU 3 was clearly related to cyanobacteria, it was not closely associated with any known species within the BLAST or RDP databases. Notably, a unicellular cyanobacteria, *Cyanobium* (Komárek et al. 1999), was more abundant in the unsuccessful (8%) than the unsuccessful cultivation (<1%).

**Fig. 5:**
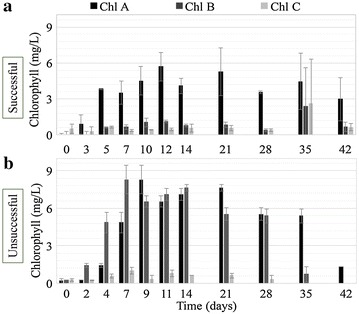
Chlorophyll *a, b*, and *c* concentrations for the successful and unsuccessful cultivation of OPGs

Only the 16S rRNA gene was considered in our sequencing analysis which did not capture eukaryotes in the analysis, so profiles of chlorophyll were also examined. During all cultivations, chlorophyll *a* increased significantly during the first two weeks of the cultivations, reaching a maximum between days 9 and 12 before a decrease in the final weeks (Fig. 5). Interestingly, the chlorophyll *a* concentration was greater during the unsuccessful cultivation at almost all time points. Chlorophyll *b* and *c* concentrations were also investigated. Chlorophyll *b* was significantly greater during the unsuccessful cultivation, indicating that a greater abundance of chlorophyll *b* producers such as algae. In the successful cultivation, this is not the case, indicating a greater abundance of phototrophs that produce chlorophyll *a* but not chlorophyll *b*, such as cyanobacteria. A greater abundance of cyanobacteria in the successful cultivation is supported by microscopy (Fig. 2) and sequencing analysis (Fig. 4).

### Functional gene abundance within the communities

**Fig. 6:**
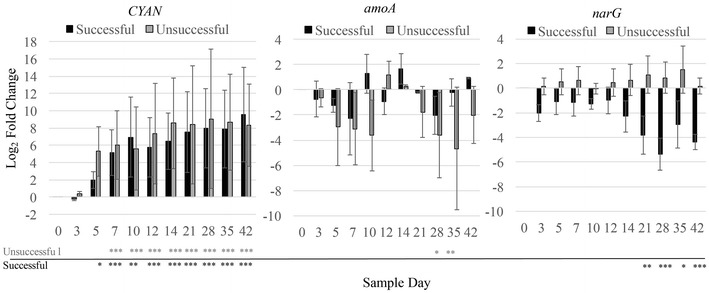
Log_2_ fold change of the target genes *CYAN*, *amoA,* and *narG* using 16S rDNA as a reference and compared to the t = 0 day community. R_2_ values were >0.98 for all calibrations. qPCR reaction efficiencies were greater than 90%. Significant interaction effects were further determined with Tukey’s HSD test if the ANOVA results were significant (p < 0.05)—*p < 0.05, **p < 0.01, ***p < 0.001)

Absence of soluble inorganic nitrogen species in the bulk liquid does not indicate absence of functionality in a community, particularly if concentrations are low and consumed quickly by microorganisms. Quantitative analysis of genomic DNA revealed the presence of genes associated with nitrification (*amoA*), denitrification (*narG*) and cyanobacteria (*CYAN*) throughout cultivation (Fig. 6; Additional file 1: S5). Consistent with trends in the chlorophyll and the sequencing data, the relative abundance of the *CYAN* (cyanobacterial 16S rRNA) gene within the communities increased for both the successful and unsuccessful cultivation (p > 0.05 or less after day 5 compared to the initial community, Figs. 5 and 6). Differences between the successful and unsuccessful cultivations in *amoA* and *narG* are more difficult to discern as vial to vial variation was high and statistical significance infrequent. For *amoA* in the unsuccessful community compared to the day 0 community, a net loss in the gene abundance is significant in the final weeks of cultivation (day 28 and day 35, Fig. 6). In a pairwise comparison, there was a significant different between day 12 and the preceding sampling days–day 5, p = 0.007, day 7, p = 0.006, day 10, p = 0.007—in the unsuccessful community, this may suggest a slight increase in the relative abundance of *amoA* during that time period. There is a net loss of *narG* in the successful cultivations towards the end of the cultivation period (day 21–42) compared to the initial community, though statistically significant trends cannot be reported for the unsuccessful cultivation. Considering the relative abundance of these three genes within a successful cultivation compared to the unsuccessful cultivation at each sampling time point suggests slightly greater relative abundance of *CYAN* in a successful cultivation and *amoA* and *narG* appear more abundant in the unsuccessful communities (Additional file 1: Figure S6). The notable exception is at day 10, when *CYAN* (2.5 log_2_ fold), *amoA* (3.2 log_2_ fold) and *narG* (0.49 log_2_ fold) are greater in the successful cultivation and likely a key time point in granulation success. The potential for nitrogen fixation was also investigated with two nitrogenase reductase (*nifH*) gene primers sets (Rösch and Bothe 2005; Segawa et al. 2014), but with negative results. The lack of amplification could indicate either a lack of nitrogen fixation or poor primer specificity for this community, though multiple qPCR assays were attempted to optimize for amplification if the genes were present.

## Discussion

Filamentous cyanobacteria are integral to the structural integrity of successful granulation (Milfrestadt et al. in review), so their early and sustained growth is crucial for a successful cultivation of OPGs. At the end of a successful cultivation, filamentous cyanobacteria comprised the large percentage of the communities with OTUs 2 and 5 associated with filamentous genera *Oscillatoria* and *Geitlernema* accounting for 46% of the relative abundance. The unsuccessful communities also had these OTUs but they accounted for a smaller percentage of the bacterial community (27%) and there was a greater abundance of an OTU associated with a unicellular cyanobacteria from the genus *Cyanobium* (8% unsuccessful vs. 1% successful).

Different groups of cyanobacteria are known to assimilate a variety of nitrogen sources, which should make their growth and abundance resilient in environments with varying concentrations and species of nitrogen. Some cyanobacteria can assimilate nitrogen via dinitrogen gas, nitrate, or urea with ammonium as a cellular intermediate or ammonium directly (Berman and Chava 1999; Flores and Herrero 2005). However, the data in this study suggests that there is a favorable relationship between the availability of inorganic nitrogen at the start of a cultivation, the abundance of filamentous cyanobacteria, and the ultimate success of that OPG formation.

The decrease in ammonium concentration in the first few days of a successful cultivation combined with an increased *CYAN* relative abundance, and increase in chlorophyll *a* within the microbial community may suggest that ammonium was assimilated in cyanobacterial growth. Phototrophic growth was also observed during this time period in an unsuccessful cultivation, though the greater increase in chlorophyll *b* concentrations may suggest that the growth also included an abundance of microalgae. Previous studies have suggested the concentration and species of inorganic nitrogen (ammonium and nitrate) influence competition between microalgae and cyanobacteria (Agawin et al. 2007; Hyenstrand et al. 2000). At ammonium concentrations similar to those in this study, the cyanobacteria, *Synechococcus,* outcompeted the microalgae, *Scenedesesmus,* because of its ability to rapidly uptake ammonium from solution (Hyenstrand et al. 2000). Similarly, with sufficient supply of ammonium or nitrate, cyanobacteria, *Cyanothece,* out grew *Chlorella* in batch experiments (Agawin et al. 2007). The results from these studies of co-cultures may inform the observations in these mixed community cultivations. The cultivations that started with greater inorganic nitrogen may lead to a greater abundance of cyanobacteria in the final populations and a greater likelihood of successful granulation of OPGs. Cyanobacteria and microalgae are not the only organisms competing for inorganic nitrogen. Ammonia-oxidizing bacteria (AOB) and archaea (AOA) and denitrifying organisms will also compete for the limited inorganic nitrogen. Evidence of these functions within community is indicated by the presence of *amoA* at all time points in both the successful and unsuccessful cultivations. When comparing the relative abundance of *amoA* in the initial community of the successful cultivation to the unsuccessful community (Additional file 1: Figure S6), there is 1.69 log_2_ fold greater representation of *amoA* in the unsuccessful cultivation. There is a precedent in marine environments that AOB/AOA are able to outcompete cyanobacteria for ammonium (Boyett et al. 2013), which may suggest that a greater initial abundance of AOB/AOA initially may preclude sufficient cyanobacterial growth for successful cultivation.

The role of denitrification in the success of OPG formation is less clear. Denitrification is a ubiquitous function among many heterotrophic bacteria and potential for the function within a community does not necessarily suggest activity within the community. However, the lack of a significant trend in relative abundance of *narG* gene in an unsuccessful cultivation and decreasing relative abundance in the successful cultivation compared to the initial microbial community implies an important difference between the two cultivation sets. Additionally, gene copy numbers suggest a net loss of this gene within the successful community (t = 0 days, 3.6 × 10^9^ ± 4.5 × 10^8^ copy numbers; t = 42 days, 1.7 × 10^9^ ± 3.7 × 10^8^ copy numbers, p < 0.0001). Nitrate concentrations were consistently low throughout cultivations for the successful cultivation making it difficult to determine if the decrease in abundance was due to lack of available nitrate, competitive nitrate assimilation by phototrophs, inhibition of denitrification by oxygen produced by phototrophs, or some combination of these factors.

Static cultivation of OPGs is a long process [42 days in this study, 21–60+ in other studies (Milfrestadt et al. in review)]. Systematic observations of successful and unsuccessful cultivations indicate that there are early indicators for successful cultivation. Likewise, these indicators could be used to monitor the health and progression of granular biomass within operating OPG reactors. Availability of inorganic nitrogen and increasing trends in relative abundance of *CYAN*, decreasing trends in *narG* relative abundance are observed in successful cultivations. Between days 10–12, there appears to be notable changes in ammonium and organic nitrogen concentration. Since the VSS concentrations remain relatively constant throughout cultivation, the increase in organic nitrogen and ammonium and increase in cyanobacterial 16S rRNA genes (CYAN) around day 10–14 may suggest a decay of the initial community in favor of new phototrophic growth. This trend is not as pronounced in the unsuccessful cultivation. While trends in abundance of different genes are good indicators of granulation success, they may not be rapid or accessible enough for monitoring an OPG cultivation or reactor operation. Chlorophyll measurements, particularly chlorophyll *a* and *b,* may be a better early indicator for relative abundance of microalgae and cyanobacteria within OPG communities. Though chlorophyll *a* concentrations were similar in both cultivations, increased concentrations of chlorophyll *b* appeared by day 2 in an unsuccessful cultivation (unsuccessful = 1.5 mg/L, successful = 0 mg/L, Fig. 5).

Cyanobacteria have been considered for wastewater treatment because of their ability to flocculate (Chevalier et al. 2000) and more recently, have been described in granules with a hybrid of phototrophs, heterotrophs, and autotrophs (Kumar and Venugopalan 2015; Milferstedt et al. in review). This work reinforces previous studies that filamentous cyanobacteria are critical for successful OPG formation as they are important to the structural rigidity of the granule. *CYAN*, *amoA* and *narG* exist in all communities which is a good indicator for the organic and nitrogen removal in wastewater treatment that has been observed by Abouhend et al. (2016). The assimilation of ammonium and nitrate by phototrophs within OPG process should be considered an important pathway in nitrogen removal. The nitrogen cycling within communities is complex; however, this study implicates initial inorganic nitrogen availability is important for the growth of filamentous cyanobacteria, likely providing them a competitive advantage in the mixed community. These filamentous cyanobacteria have been shown to be critical to OPGs (Milferstedt et al. in review) and sustaining their abundance within reactors will be an important parameter for success of OPG reactors used for wastewater treatment.

## Additional file

**Additional file 1.** The supporting information is available. It contains additional details regarding the trends of data described in the text. The Supporting information contains figures that include: Primer information (Table S1), Phosphate (Figure S1), soluble COD (Figure S2), Suspended Solids (TSS/VSS) (Figure S3) trends referenced in this manuscript. Additionally, data supporting the microbial ecology and gene abundance is presented as a heat map of the top 20 OTUs for the successful and unsuccessful communities at the genus level for times 0 days and 42 days (Figure S4), the absolute gene copy numbers for *CYAN*, *amoA*, and *narG* (Figure S5), and the log_2_ fold change of the target genes *CYAN*, *amoA,* and *narG* using 16S rDNA as a reference for the successful community and compared to the unsuccessful community for each time point (Figure S6).

## Abbreviations

AOA: ammonium oxidizing archaea
AOB: ammonium oxidizing bacteria
COD: chemical oxygen demand
OPG: oxygenic photogranule
OTU: operational taxonomic unit
TSS: total suspended solids
VSS: volatile suspended solids
WWTP: wastewater treatment plant
