# Supplementary material for: The role of inorganic nitrogen in successful formation of granular biofilms for wastewater treatment that support cyanobacteria and bacteria
Source: AMB Express. 2017 Jul 10;7:146. doi: 10.1186/s13568-017-0444-8 (PMC5503847; doi:10.1186/s13568-017-0444-8)
Supplement: Supplementary file 1 — Additional file 1. The supporting information is available. It contains additional details regarding the trends of data described in the text. The Supporting information contains figures that include: Primer information (Table S1), Phosphate (Figure S1), soluble COD (Figure S2), Suspended Solids (TSS/VSS) (Figure S3) trends referenced in this manuscript. Additionally, data supporting the microbial ecology and gene abundance is presented as a heat map of the top 20 OTUs for the successful and unsuccessful communities at the genus level for times 0 days and 42 days (Figure S4), the absolute gene copy numbers for CYAN, amoA, and narG (Figure S5), and the log2 fold change of the target genes CYAN, amoA, and narG using 16S rDNA as a reference for the successful community and compared to the unsuccessful community for each time point (Figure S6). [file 13568_2017_444_MOESM1_ESM.pdf]

Applied Microbiology and Biotechnology

**The role of inorganic nitrogen in successful formation of granular biofilms for wastewater treatment that support cyanobacteria and bacteria**

Kristie Stauch-White, Varun N. Srinivasan, W. Camilla Kuo-Dahab, Chul Park, and Caitlyn S. Butler*
Department of Civil and Environmental Engineering, University of Massachusetts, Amherst, 01003,
*corresponding author: cbutler@ecs.umass.edu, 413-545-5396

## Supporting Information:

This Supporting information contains expansion of data referenced in the text of manuscript. These figures and tables that include: Primer information (Table S1), Phosphate (Figure S1), soluble COD (Figure S2), Suspended Solids (TSS/VSS) (Figure S3) trends referenced in this manuscript. Additionally, data supporting the microbial ecology and gene abundance is presented as a heat map of the top 20 OTUs for the successful and unsuccessful communities at the genus level for times 0 days and 42 days (Figure S4), the absolute gene copy numbers for Cyanobacterial 16S rRNA (*CYAN)*, *AmoA*, and *narG* (Figure S5), and the $\log_2$ fold change of the target genes *CYAN*, *amoA,* and *narG* using 16S rDNA as a reference for the successful community and compared to the unsuccessful community for each time point (Figure S6).

Table 1.

Table S1. Primers used in this study for PCR and qPCR analysis.

| Target Gene Name or Shorthand | Target Description | Primer Name | Sequence (5' – 3') | Anneal (°C) | Length (bp) | References |
|---|---|---|---|---|---|---|
| *amoA* | *Betaproteobacteria* ammonia monoxygenase | amoA-1F<br>amoA-2R | GGGGHTTYTACTGGTGGT<br>CCCCTCKGSAAAGCCTTCTTC | 58 | 491 | (Rotthauwe, Witzel, & Liesack, 1997; Segawa et al., 2014) |
| *narG* | Nitrate reductase | narG-F<br>narG-R | TCGCCSATYCCGGCSATGTC<br>GAGTTGTACCAGTCRGCSGAYTCSG | 60 | 173 | (Bru, Sarr, & Philippot, 2007) |
| Cyanobacteria 16S rRNA (CYAN) | Cyanobacteria 16S rRNA gene | CYAN 108F<br>CYAN 377R | ACGGGTGAGTAACRCGTRA<br>CCATGGCGGAAAATTCCC | 52 | 269 | (Martins & Vasconcelos, 2011; Urbach, Robertson, & Chisholm, 1992) |
| 16S rRNA gene | Universal 16S | 1114<br>1275 | CGGCAACGAGCGCAACCC<br>CCATTGTAGCACGTGTGTAGCC | 60 | 161 | (Denman & McSweeney, 2006) |

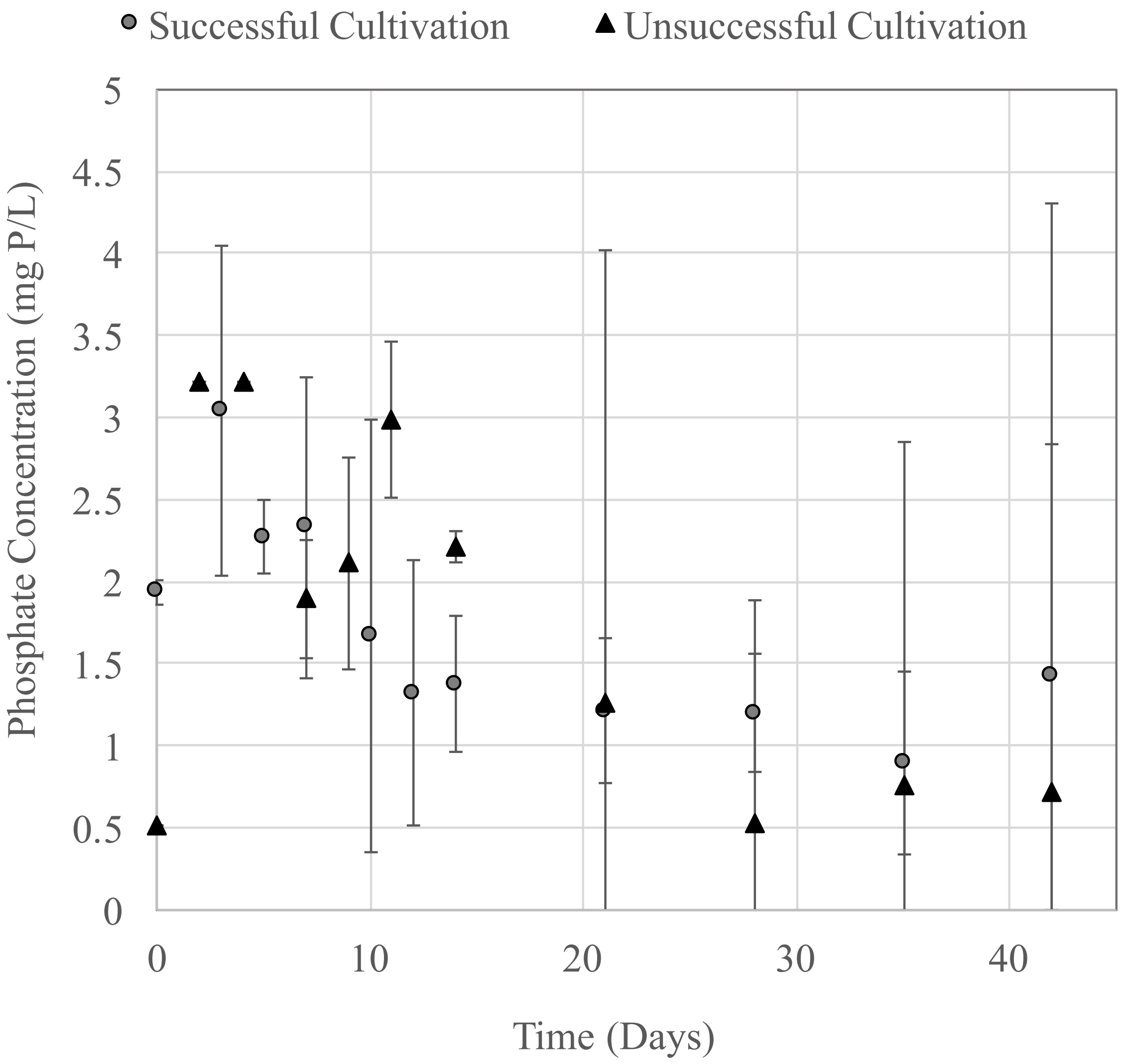


**Figure S1.** Phosphate concentration for successful and unsuccessful cultivations

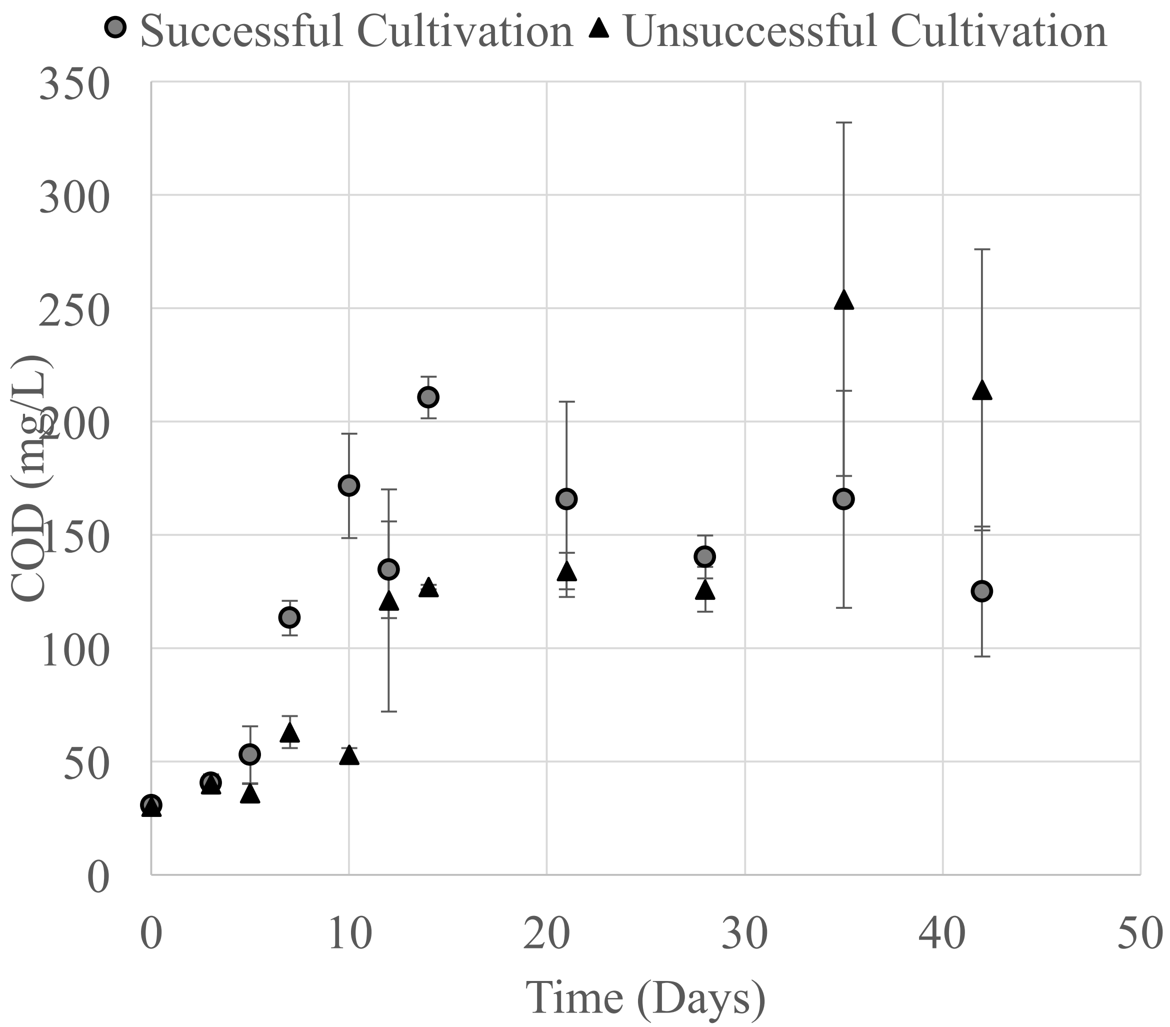


**Figure S2.** Soluble COD concentration for successful and unsuccessful cultivations

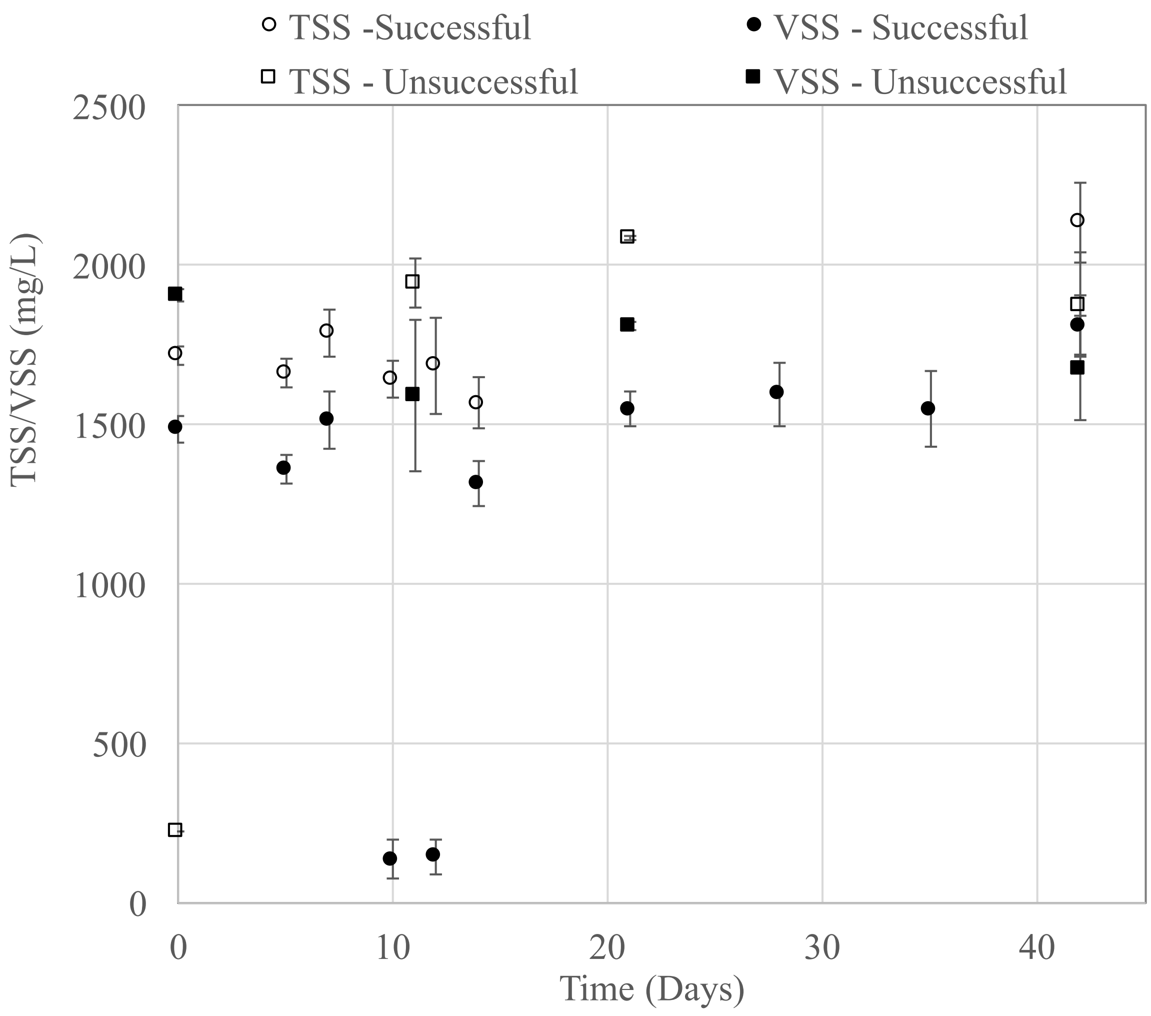


**Figure S3.** TSS and VSS concentration for successful and unsuccessful cultivations

**Figure S4.** Heat map for the top twenty OTUs for the initial and final communities at times 0 and 42 days.

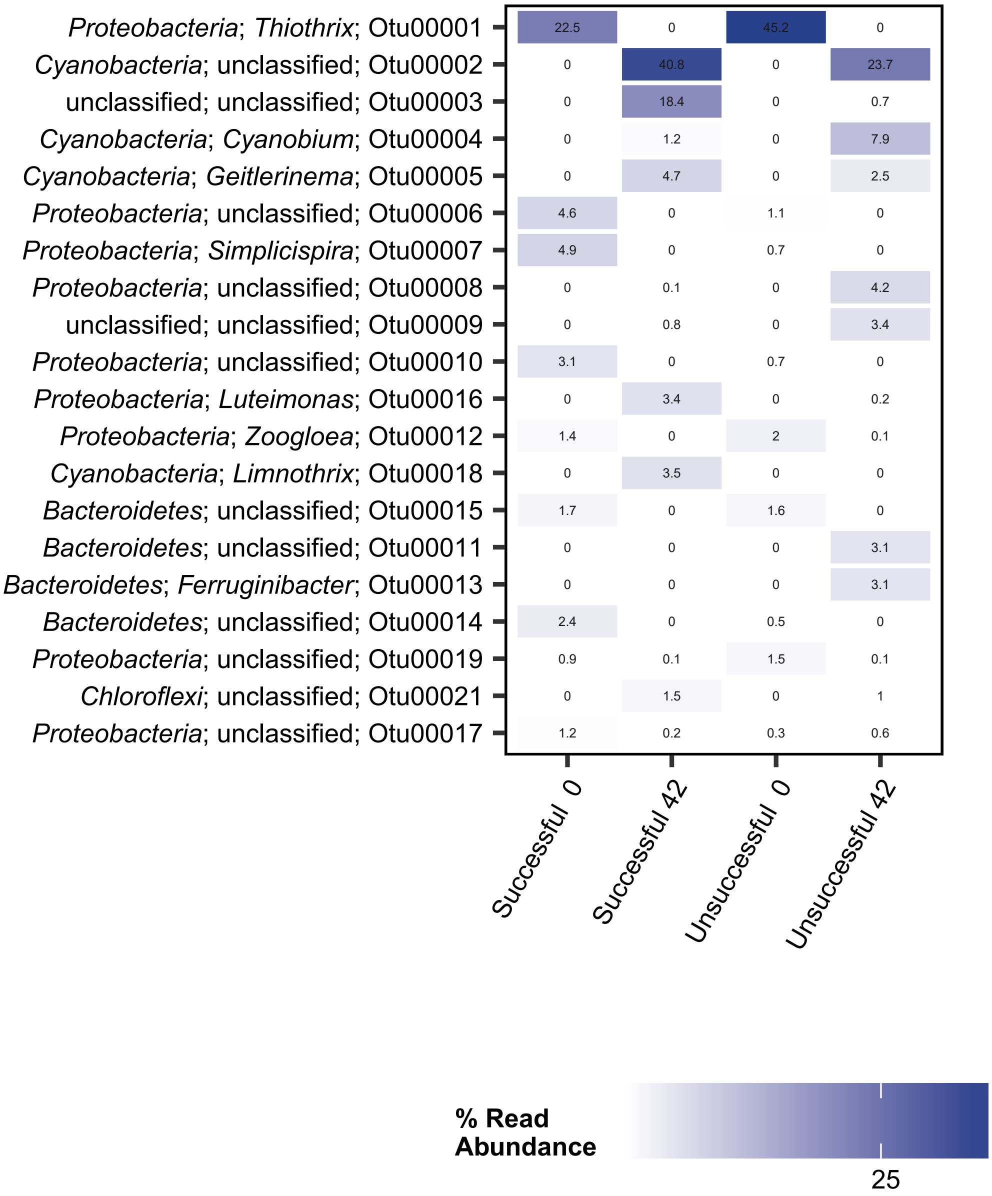

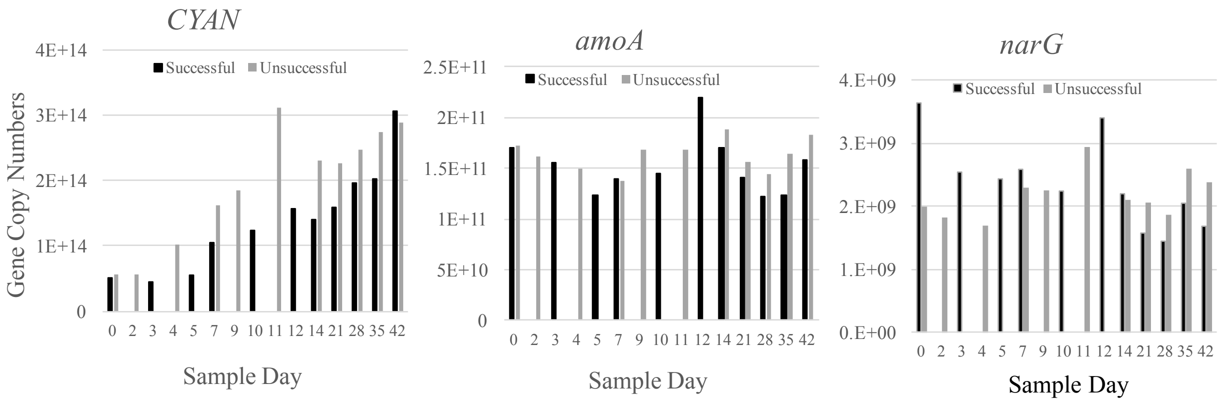


**Figure S5.** Gene Copy numbers of the target genes *CYAN*, *amoA,* and *narG* for the successful community and unsuccessful community for each time point. Absolute copy numbers were calculated using standard curves constructed qPCR reactions

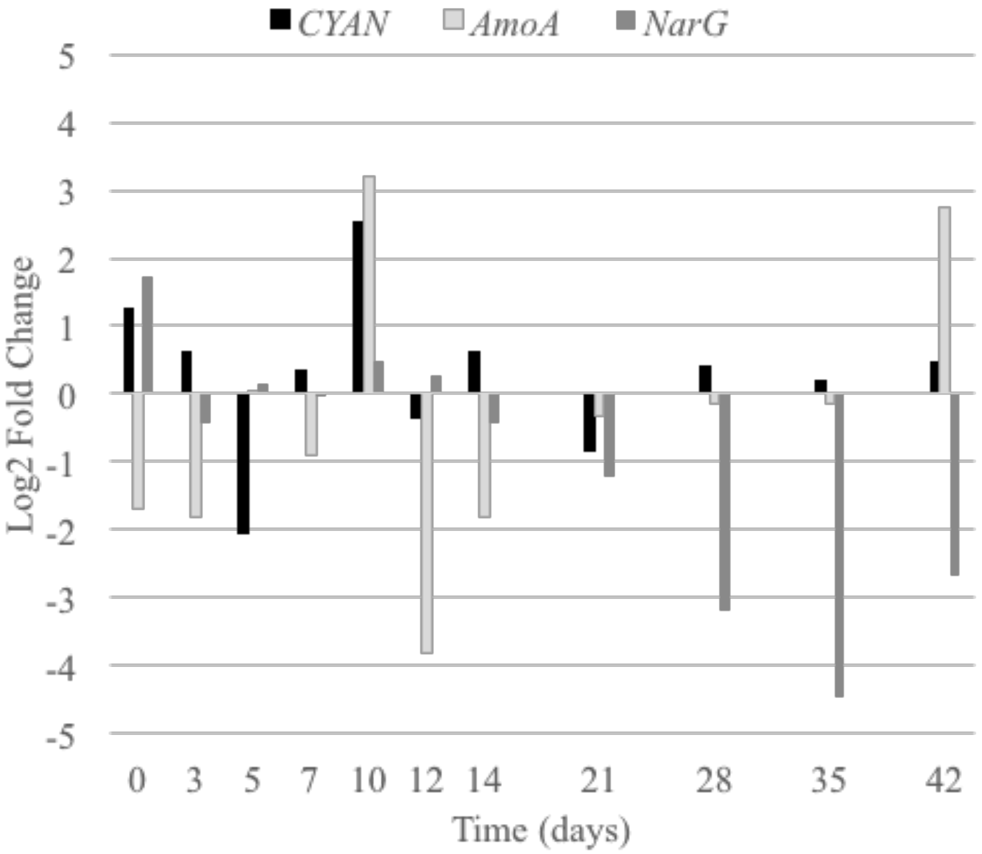


**Figure S6.** $Log_2$ fold change of the target genes *CYAN*, *amoA,* and *narG* using 16S rDNA as a reference for the successful community and compared to the unsuccessful community for each time point. $R_2$ values were > 0.98 for all calibrations and reaction efficiencies were greater than 90%
